#  Mechanism of PhosphoThreonine/Serine Recognition and Specificity for Modular Domains from All-atom Molecular Dynamics

**DOI:** 10.1186/2046-1682-4-12

**Published:** 2011-05-25

**Authors:** Yu-ming M Huang, Chia-en A Chang

**Affiliations:** 1 Department of Chemistry, University of California, Riverside, CA92521, USA

## Abstract

**Background:**

Phosphopeptide-binding domains mediate many vital cellular processes such as signal transduction and protein recognition. We studied three well-known domains important for signal transduction: BRCT repeats, WW domain and forkhead-associated (FHA) domain. The first two recognize both phosphothreonine (pThr) and phosphoserine (pSer) residues, but FHA has high specificity for pThr residues. Here we used molecular dynamics (MD) simulations to reveal how FHA exclusively chooses pThr and how BRCT and WW recognize both pThr/pSer. The work also investigated the energies and thermodynamic information of intermolecular interactions.

**Results:**

Simulations carried out included wide-type and mutated systems. Through analysis of MD simulations, we found that the conserved His residue defines dual loops feature of the FHA domain, which creates a small cavity reserved for only the methyl group of pThr. These well-organized loop interactions directly response to the pThr binding selectivity, while single loop (the 2nd phosphobinding site of FHA) or in combination with α-helix (BRCT repeats) or β-sheet (WW domain) fail to differentiate pThr/pSer.

**Conclusions:**

Understanding the domain pre-organizations constructed by conserved residues and the driving force of domain-phosphopeptide recognition provides structural insight into pThr specific binding, which also helps in engineering proteins and designing peptide inhibitors.

## Background

Protein phosphorylation is widely exploited in DNA damage repair, signal transduction, cell growth and cell cycle regulation; the cascades of downstream signals can be triggered by grabbing a certain phosphoprotein [1-6]. Elucidating the characteristics of phosphopeptide recognition is fundamental to study cellular functions [7]. The phosphoproteins are usually classified into two families, phosphotyrosine (pTyr)-containing and phosphoserine (pSer)/phosphothreonine (pThr)-containing sequences which are phosphorylated and dephosphorylated by different categories of kinases (e.g., pThr/pSer kinase and pThr kinase) and phosphatases [8]. Recent studies discovered a few modular domains that particularly recognize pThr/pSer- or pThr-containing sequences, such as the breast-cancer-associated protein BRCA1 C-terminal (BRCT) repeats, WW domain and forkhead-associated (FHA) domain [9]. Among them, the FHA domain differentiates pThr-containing peptides from pSer-containing peptides, although the difference is only one methyl group [10-15]. Because Ser/Thr kinase phosphorylates both residues, the FHA domain can efficiently reduce potential interaction sites by specifically binding to pThr-containing regions. Although experimental structures and recent studies have shown important interactions involve in the binding of the methyl group of pThr [14,16], the detailed mechanisms of the phosphoresidue recognition of different domains and how the FHA domain can reserve non-polar interactions for a small non-polar methyl group are not fully understood.

The FHA domain is associated with proteins of diverse functions in different organisms. For example, the Rad53-FHA1 domain interacts with phosphorylated Rad9 in response to DNA damage, and the Dun1-FHA domain interacts with SCD1 of Rad53, which leads to activation of Dun1in response to DNA damage response [17]. Instead of binding to a single pThr, the formation of the Dun1-FHA:SCD1 complex requires two phosphoresidues, which suggests a potential mechanism whereby sequential signaling events could be triggered through the recognition of multiple phosphoresidue-binding sites. Similar to Dun1-FHA, Ki67-FHA domain also recognizes dual phosphorylated residues at the same time [18], and the sequence identities of Dun1-FHA and the Ki67-FHA to Rad53-FHA1 are both 34%.

**Figure 1:**
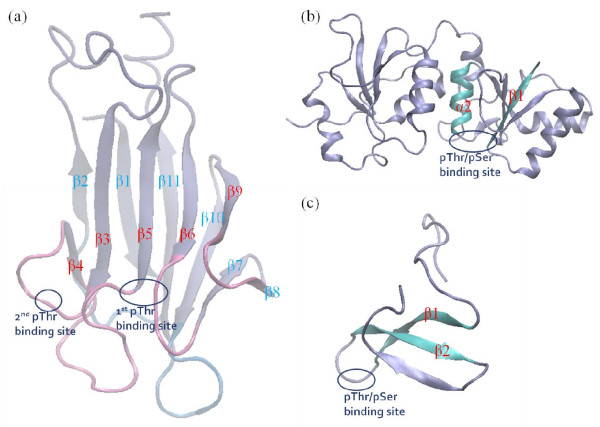
**Overall architecture of signaling domains**. (a) Snapshot of Rad53-FHA1 molecular dynamics (MD) simulations. Pink and blue represent front and back loop/β-strand respectively. (b) Snapshot of an MD simulation for BRCT repeats in BRCA1. (c) WW domain in Pin1 protein.

The structure of the FHA domain is mostly organized into a twisted β sandwich of 11 well-defined β sheets, five in the front and six at the back (Figure 1 (a)) [12,14,16,19-23]. The domain contains ~120 to 140 residues, but only five to ten residues are conserved. Six loops connected to the secondary β strands constructing the pThr binding site are the main difference between distinct FHA domains. Experimental structures show that the synthetic peptides bind to the loops between β3-β4, β4-β5, β6-β7 and β10-β11, and the conserved pThr binding site locates between loops β4-β5 and β6-β7 (Figure 1 (a)) [12,14].

BRCT repeats in BRCA1 are considered to be related to breast cancer [24-27]. The structure of the tandem BRCA1-BRCT repeats bind to phosphorylated protein that contains pSer or pThr, although binding to pSer is preferred [28]. Several structural studies have revealed a conserved structure for the repeats, mainly composed of α helixes, β sheets and loops that link secondary structures. The phosphoresidue-recognized site is located between β1 and α2 (Figure 1 (b)) [29-31].

The WW domain in Pin1 is essential for mitotic progression [32]. The domain has only about 40 residues and is one of the smallest pThr/pSer binding domains [3]. It specifically binds to pThr-Pro- or pSer-Pro-containing motifs with slightly higher affinity for pThr-Pro-containing peptides [33]. For example, in the Pin1-WW domain, the aromatic rings of Tyr23 and Trp34 define a steric clamp to confer a Pro adjacent to pSer/pThr [34]. The WW domain folds into three anti-parallel β stands and Arg21 and Ser22 residues in the loops between β1 and β2 are the phosphate group recognition sites (Figure 1 (c)) [3,4,34].

Because FHA domains are pThr specific modular domains, this study focused on how FHA domains display selective for pThr/pSer residues and comparison with BRCT and WW domains. We study the dynamic and conformational changes of the free domain and the complexes of Rad53-FHA1:Rad9, Dun1-FHA:SCD1, Ki67-FHA:hNIFK, BRCA1-BRCT:BRCH1 and Pin1-WW:CTD systems. We also computed the interaction energy between pThr/pSer and the domains to disclose the driving force of pThr/pSer binding. We propose a model for pThr specificity and potential applications.

## Methods

### Molecular systems

We selected three FHA domains in different families, BRCT repeats and the WW domain. One of the FHA domains is the human Chk2 homolog in yeast, Rad53-FHA1, involved in checkpoint signaling in *Saccharomyces. Serevisiae*. The target protein of Rad53, Rad9, is phosphorylated in response to DNA damage and interacts with the C-terminal FHA1 domain of Rad53. Two initial structures are from crystallographic coordinates (Protein Data Bank (PDB) code 1G6G) [12] and NMR structure (PDB code 1K3Q) [16]. Both Rad53-FHA1 domains share the same protein sequence. Another FHA domain is from the Dun1 checkpoint kinase. The Dun1-FHA domain interacts with phosphorylated SCD1 of Rad53, which leads to activation of Dun1. The Dun1-FHA and SCD1complex of the domain-peptide structure is acquired from the PDB code 2JQL[35]. The other system of the FHA domain near the N-terminus of human Ki67 antigen protein that interacts with human nucleolar protein hNIFK was studied a few years ago. The structural complex of Ki67-FHA and a 44-residues fragment in phosphorylated hNIFK is explored by the coordinates of the PDB code 2AFF[18]. We chose to study the pThr/pSer binding modular domain (PDB code 1T2V) of the complex of the BRCT domain in the BRCA1 C-terminus and its target binding partner, BRCH1 [29]. Another phosphodomain, the WW domain from the Pin1 N-terminus, interacts with the heptaphophorylated peptide in the CTD domain (PDB code 1F8A) [34]. Although the peptide contains two pSer residues, only one binds to the domain. All peptide sequences are in Table 1. To study the recognition for phosphoresidue and how FHA domains differentiate the pThr/pSer residue, we manually mutated pThr to pSer or pSer to pThr in phosphopeptides. The mutated sites are shown in Table 1.

**Table 1:** Peptide sequences of domain-phosphopeptide complexes

domain	Protein	PDB ID	Method	Phosphopeptide	Kd(μM)	**Ref**.
**FHA1**	**Rad53**	1G6G	X-ray	LEV**(pT)**EADATFAK	0.53	(12)
**FHA1**	**Rad53**	1K3Q	NMR	SLEV**(pT)**EADATFVQ	0.3	(16)
**FHA**	**Dun1**	2JQL	NMR	NI(pT)QP**(pT)**QQST	0.3-1.2	(35)
**FHA**	**Ki67**	2AFF	NMR	KTVD(pS)QGP**(pT)**PVC(pT)*PTFLERRKSQVAE*LNDDDKDD*EIVFK*QPISC	0.077	(18)
**BRCT**	**BRCA1**	1T2V	X-ray	AAYDI**(pS)**QVFPFA	0.4	(29)
**WW**	**Pin1**	1F8A	X-ray	Y(pS)PT**(pS)**PS	34	(34)

Residues in the bracket are either pT or pS. The first mutation site is represented by both underline and bold, and the second mutation site is underlined. Secondary structures, α helix and β sheet, are labeled as italic with underline and italic, respectively.

### Molecular dynamics simulation

We performed molecular dynamics (MD) simulations using the Amber10 and NAMD2.6 simulation packages with the ff99sb amber force field [36-38]. Because phosphoresidues are not defined in the ff99sb force field, we used the pThr and pSer force field developed by Homeyer *et al*. [39]. All simulations of wild-type sequences were initialed from six experimental complexes. In this work, we studied six un-mutated complexes and eight mutated structures. The protonation states were checked by the MCCE program [40]. All complexes were solvated in a rectangular box of 12 Å TIP3P water with the tleap program in Amber10 [41]. The placement of counter-ions of Na^+ ^was based on the Columbic potential to keep the whole system neutral. Particle Mesh Ewald was used to consider the long-range electrostatic interactions [42]. Following 10,000 and 20,000 steps of minimization of the water and system, respectively, the systems were gradually heated for each complex from 250 K for 20-ps, 275 K for 20-ps and 300 K for 200-ps. To initial the mutated structures, after equilibrium from 300 K, we manually added or deleted the methyl group and changed the residue name accordingly, then used the Amber program to build the prmtop files for mutants. A quick 100-steps minimization was applied to the mutants, then we preformed 20-ps equilibrium at 300 K. All MD simulations for each wild-type and mutated complex was performed in 1 ns by five different random number seeds to generate different initial velocity. The resulting trajectories were collected every 1 ps and the time step was 2-fs. The NPT ensemble was applied, and periodic boundary conditions were used throughout the MD simulations. A temperature of 300 K was maintained with use of a Langevin thermostat, with a damping constant of 2 ps^-1^, and the hybrid Nose-Hoover Langevin piston method was used to control the pressure at 1 atm. The SHAKE procedure was used to constrain hydrogen atoms during MD simulations [43].

### Binding energy calculation by MM-PBSA methods and entropy calculation

To quantify the stability of the phosphopeptide binding to the domain, we performed end-point energy calculations, also known as MM-PBSA/MM-GBSA calculations [44-50]. A simple thermodynamic cycle and single-trajectory post-processing allows for efficiently computing the various contributions to the domain-peptide binding. We used the structural ensemble obtained from the final 1-ns of each random number seed to demonstrate the post-energy calculations. The binding interaction energy, ΔE_bind_, associated with the binding of a domain to its cognate peptide to form a protein-peptide complex is calculated as follows:(1)

The bracket <E > denotes the average energy computed from a given MD trajectory. The changes in average energy on molecular interactions can be decomposed as follows:(2)

representing the changes in valance (v) energy (bond, angle, dihedral, and improper dihedral energies), van der Waal (vdw) interactions, Coulombic (Coul) interactions, and solvation free energy ΔW_PB_/ΔW_GB _and ΔW_np_. We note that the binding energy computed here includes the solvation free energy which considers water entropy, and the valance energies cancel out in Eq. 2 due to the single trajectory approach. The solvation free energy can be further decomposed into the polar term, ΔW_PB_/ΔW_GB_, and non-polar cavity term, ΔW_np _term [44,51]. Here, we demonstrate two methods, Poisson-Boltzmann (PB) and Generalized-Born (GB), to estimate the polar solvation term [45,46]. PB was calculated by solving the PB equation in the PBSA model of Amber11. The dielectric constants of the interior and exterior protein were set to 1 and 80 respectively. GB (igb = 1) was used in the sander program of Amber11 package. The non-polar solvation term was calculated by the solvent-accessible surface area (SASA) model. All energy involved a 40 Å cutoff value for non-bonded interactions.

The configuration entropy S consisted of phi, psi, omega and sidechain dihedrals, which include both conformational (number of energy wells) and vibrational entropy (width of an energy well) [47,48,52]. We computed each dihedral angle entropy using the Gibbs entropy formula:(3)

where p(x) is the probability distribution of dihedral x, and R is the gas constant. T-analyst was used to compute the Gibbs entropy [53]. We considered only the internal dihedral degree of freedom of each dihedral, and the coupling between dihedrals was ignored. The change in configuration entropy during the mutation can be presented as follows:(4)

where X denotes each dihedral angle, such as phi, psi, omega and sidechain.

## Results and discussion

Ser and Thr have very similar sidechains, so the Ser/Thr protein kinases phosphorylate the OH group of either residue without discriminating between them. Similarly, most pSer/pThr binding modules, such as BRCT and WW domains, can specifically bind to short pSer/pThr-containing motifs equally well. Not unsurprisingly, one more methyl group of Thr has few effects on the overall binding and molecular recognition. However, uniquely, most FHA domains recognize only pThr residues in target proteins, and substitution of pSer for pThr in model peptides severely weakens binding. The pThr specific recognition may function as a filter to further select the protein partner.

One of our aims was to investigate how FHA domains can distinguish the tiny difference between Ser and Thr in their binding partner. The work helps gain deeper understanding in molecular recognition and provides valuable insight into strategies of protein engineering. Although we have simulated the entire FHA, WW and BRCT systems, we do not attempt to draw conclusions on properties of the whole system. Instead, we focus on the phosphoresidue binding region, in particular pThr or pSer. Multiple 1 ns MD simulations were performed for each system with different initial velocity so that the simulations evolve independently of each other to reduce potential bias caused by the initial conformation. Because the short phosphopeptides are highly flexible, carrying out short simulations allows the peptide to stay in similar local energy well during different runs [54,55]. The root mean square deviation (RMSD) of selected systems is shown in Figure S1 of Additional file 1.

To obtain an estimate of the differences in interaction energies of the domain with its pSer- or pThr-containing peptides, we post-processed our MD trajectories and computed the domain-peptide interaction energies, including the potential energy and solvation energy. The method is usually called the MM-PBSA method. We computed intermolecular interactions between the whole domain and the entire peptide, termed the "global binding energy calculation". In our global energy calculations, the solvation energy term includes a PB term, W_PB_, for electrostatic solvation free energy, and a cavity/surface area term, W_np_, for nonpolar solvation free energy. Because we are particularly interested in studying the local region that contributes significantly to pThr or pSer recognition, we also selected residues within 5 to 7 Å of the phosphate group (Table S3 in Additional file 1) to calculate interaction energies between the residues chosen. We called the calculations between these selected residues "local interaction energy calculation". Because the calculations involved only residues near the binding sites, the solvation energies computed by the PB or GB model are similar. As a result, we used the GB model in our local energy calculation to speed up the calculation.

### Revealing the specificity of the main pThr-binding site in FHA domains

**Figure 2:**
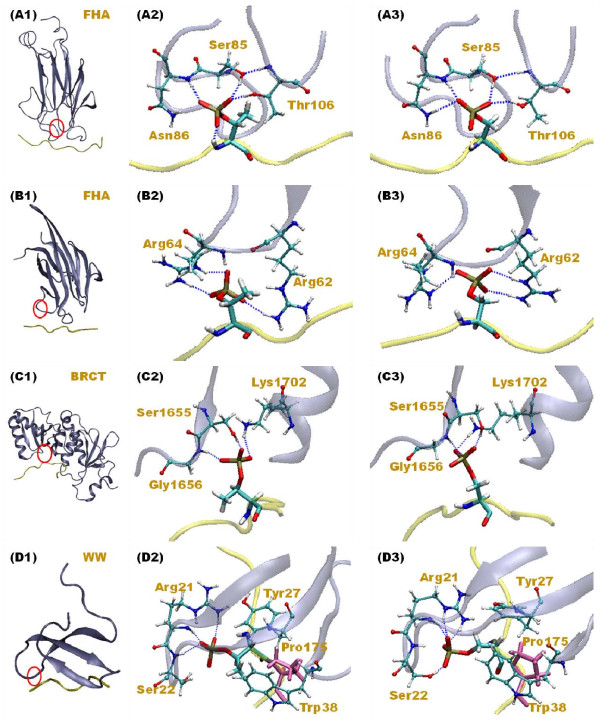
**Detailed illustration of pThr binding in Rad53-FHA1 main binding site (A), Dun1-FHA second binding site (B), BRCT repeats (C) and WW domain (D)**. The binding areas are circled in red (see (1) on the left side). Residues surrounding pThr and pSer residues are in (2) and (3), respectively. Atom pairs that have charge interactions with phosphoresidues are shown with a blue dashed line. Figures are a snapshot of our MD simulations.

In the FHA domain family, the loops that link two β-sheets appear to play a pivotal role in constructing the binding pocket for pThr-containing peptides. Despite the variability of primitive sequences in the loop area in different FHA-containing proteins such as Rad53-FHA1, Dun1-FHA and Ki67-FHA, analysis of FHA domain conformations suggests a conserved structure in the main pThr-binding site formed by two loops between β3-β4 and β6-β7 (see Figure 2 (A1)).

We substituted pThr with pSer *in silico*, and the global binding energy calculations show that pSer-containing peptides have 3-6 kcal/mol higher binding energies than the pThr-containing peptide. Notably, the binding energy calculations are for potential (MM) and solvation (PB/SA) energy only, and the entropic changes upon binding are not included here. Of interest is knowing which energy term contributes more to weaken the FHA domain-peptide interaction. Because the only difference between pThr and pSer is one non-polar methyl group, the pSer-containing peptide may reasonably result in less favorable van der Waals attraction. However, the trend is not clear, which suggests that replacing pThr by pSer may affect interactions between pThr/pSer and the domain, and the stability of the entire peptide binding to the protein. For example, pSer-containing peptides have weaker van der Waals interactions between Rad53-FHA1 and Ki67-FHA (Table 2), but the interaction is in the opposite direction for Dun1-FHA. Moreover, the Ki67-FHA:pSer-peptide complex shows slightly more favorable electrostatic attraction (-0.98 kcal/mol) as compared with the Ki67-FHA1:pThr-peptide complex. However, the Rad53-FHA1:pSer-peptide and Dun1-FHA1:pSer-peptide complexes have weaker electrostatic interactions. Of note, the polar contributions from the solvation model (PB term) are mostly compensated with the Coulombic term. Therefore, although all FHA:pSer-peptide complexes can form more stable Coulombic interactions, they also result in less stable solvation energy (PB term). In summary, in considering all energy terms, pThr-containing peptides are still highly favored, and our results are in good agreement with other experiments [12,16].

**Table 2:** Global MM-PBSA energy calculations

domain	mutated site	mutation	**ΔΔU**_**VDW**_	**ΔΔU**_**Coul**_	**ΔΔW**_**PB**_	**ΔΔE**_**ele**_	**ΔΔE**_**tot-np**_	**ΔΔW**_**np**_	**ΔΔE**_**tot**_
**Rad53-FHA1**	1	**pT**→**pS**	2.50	-34.34	37.83	3.49	5.99	0.75	6.74
**Rad53-FHA1**	1	**pT**→**pS**	0.44	-13.74	16.08	2.33	2.77	0.72	3.49
**Dun1-FHA**	1	**pT**→**pS**	-2.63	-13.70	18.84	5.15	2.52	2.34	4.86
**Ki67-FHA**	1	**pT**→**pS**	4.30	-8.77	7.79	-0.98	3.32	-0.84	2.48
**Dun1-FHA**	2	**pT**→**pS**	-2.54	-59.30	63.39	4.09	1.55	1.95	3.50
**Ki67-FHA**	2	**pS**→**pT**	0.94	19.77	-20.15	-0.38	0.55	0.36	0.91
**BRCT**	1	**pS**→**pT**	-3.33	28.77	-23.67	5.10	1.77	0.84	2.61
**WW**	1	**pS**→**pT**	-1.03	-39.75	36.78	-2.96	-3.99	-0.01	-4.00

ΔΔU_Coul _and ΔΔU_vdw _are the electrostatic and van der Waals interactions, respectively, between the wild-type and mutants; ΔΔW_PB _and ΔΔW_np _are the polar and non-polar contributions from the solvation energy. ΔΔE_ele _represents the sum of ΔΔU_Coul _and ΔΔW_PB_. ΔΔ indicates the changes between two calculations of the mutated and non-mutated state. For example, ΔΔU_VDW _= ΔU_VDW,pSer_-ΔU_VDW,pThr_, where ΔU_VDW _is the interaction energy between the phosphopeptide and the domain.

We also performed local interaction energy calculations and focused on the interactions between pSer/pThr and residues around the phosphoresidue to reveal how FHA can discriminate between them. Although the only difference between the Thr and Ser residue is one methyl group, which is usually considered not significant, our study indicates that the methyl group directly interacts with residues of loops β4-β5 and β6-β7 of the FHA domain (see Figure 2 (A2) and (A3)). Again, the trend agrees with the global binding energy calculations, and the local interaction energy is less favorable when pThr is replaced by pSer. The local interaction energy calculations show that van der Waals interactions are weakened considerably by the lack of a single methyl group of pSer; the loss of the van der Waals attraction can be weakened by ~3 kcal/mol (Table 3). The interaction between the methyl group of pThr and the nearby residues are unlikely to be 3 kcal/mol, but instead, the computed energy reveals the crucial role of the methyl group to stabilize the complex conformation locally. Interestingly, although the phosphate group of pSer still retains hydrogen bonding between the nearby residues of FHA, the electrostatic attractions are still weakened. This again supports that solely forming H-bonds between the phosphate group of the phosphoresidue is not enough for phosphopeptide and FHA domain binding, and lacking the methyl group destabilizes the complex. As illustrated in Figure 1 (A2) (A3) and Figure S2 in Additional file 1, fewer contacts can be formed when pSer is present in the peptide.

**Table 3:** Local MM-PBSA energy calculations

domain	mutated site	mutation	**ΔΔU**_**VDW**_	**ΔΔU**_**Coul**_	**ΔΔW**_**GB**_	**ΔΔE**_**ele**_	**ΔΔE**_**tot-np**_	**ΔΔW**_**np**_	**ΔΔE**_**tot**_
**Rad53-FHA1**	1	**pT**→**pS**	3.12	-8.36	5.71	-2.65	0.46	0.03	0.49
**Rad53-FHA1**	1	**pT**→**pS**	0.23	-9.64	10.93	1.29	1.52	-0.12	1.40
**Dun1-FHA**	1	**pT**→**pS**	2.30	3.00	-0.71	2.29	4.59	0.06	4.66
**Ki67-FHA**	1	**pT**→**pS**	3.22	2.25	-1.67	0.58	3.80	0.14	3.94
**Dun1-FHA**	2	**pT**→**pS**	0.49	-1.30	-0.26	-1.57	-1.07	0.00	-1.07
**Ki67-FHA**	2	**pS**→**pT**	0.70	3.33	-2.02	1.30	2.01	0.09	2.10
**BRCT**	1	**pS**→**pT**	0.06	7.17	-6.75	0.41	0.47	0.08	0.56
**WW**	1	**pS**→**pT**	-1.85	1.67	0.10	1.78	-0.07	0.05	-0.02

We selected residues within 5 to 7 Å around pThr/pSer residues. The residues selected are in the Additional file 1. Table S3. The notations are the same in Table 2.

Of note, although experimental structures demonstrate a pocket to accept the pThr methyl group, the static conformation cannot ascertain that pSer fails to form equally good interactions with the nearby residues, because the protein is dynamic and may fill the space by slightly changing the protein conformations. Nevertheless, our MD simulations show that the cavity is highly suited to pThr, and small changes in this particular residue can diminish the domain-peptide interactions.

**Figure 3:**
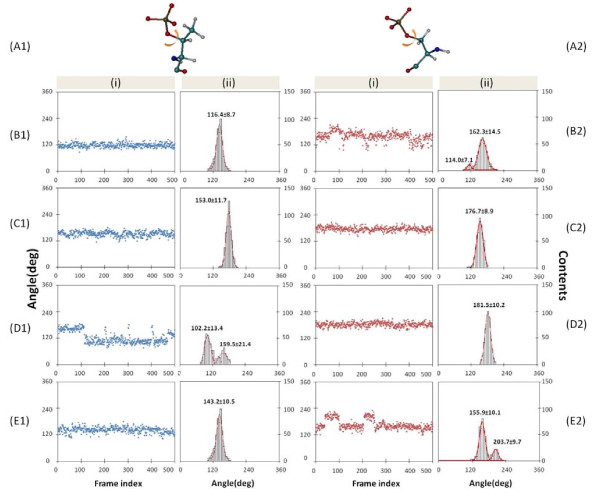
**Distribution of a dihedral angle of the phosphoresidue**. (i) Plots of the dihedral angle with five seeds are shown in frame index 1-100, 101-200, 201-300, 301-400 and 401-500, respectively and (ii) corresponding population distributions. Column (A) shows the dihedral angle of pThr (1) or pSer (2) used for plotting. (B), (C), (D) and (E) indicate the binding area in the main binding site of FHA, the second binding site of FHA, the pThr/pSer binding site of BRCT repeats and the WW domain, respectively.

**Figure 4:**
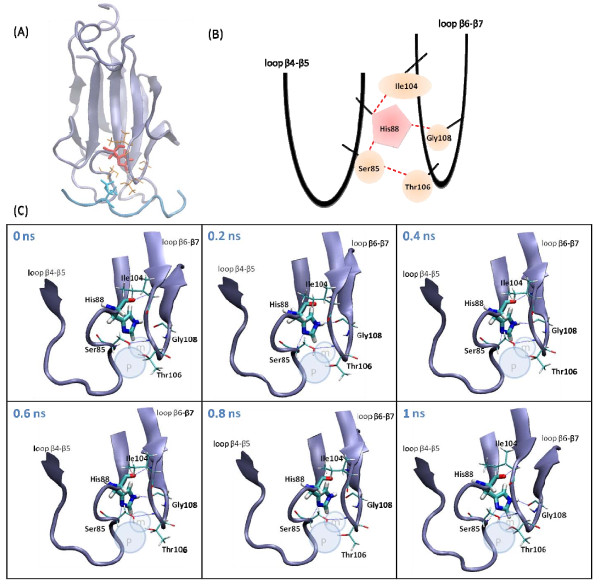
**Detailed illustration of conserved His interactions in Rad53-FHA1**. (A) Overall FHA structure. Conserved His is in red; other residues around His that contribute to form a pThr binding pocket are in orange. The phosphopeptide and pThr are in cyan. (B) Cartoon representation of two loops held by His and the nearby residues. Red dashed lines indicate interactions between residues. (C) MD simulation snapshots with time. Atom pairs with electrostatic attractions are labeled with blue dashed lines.

Because pSer shows less perfect geometry complementary to the binding cavity of the FHA domain, we also studied whether the local flexibility is changed because of the lack of the methyl group. The rotameric states of each sidechain of phosphopeptides, as well as their configuration entropy, were calculated. Most sidechain dihedrals stay in the same rotametic states for both pSer/pThr residues, but the second pThr sidechain dihedral angle (see Figure 3 (A)) differs. Figure 3 (B1) and (B2) shows the distribution of the sidechain dihedral angle of pThr and pSer in Rad53-FHA1 peptide. The dihedral of pSer deviates from pThr with an angle shift from 115 to 162 degrees, and also has wider distribution, so the dihedral is more flexible. Clearly, the methyl group of pThr allows the phosphoresidue to fill the entire pocket of the binding site, and no room is available for spacious vibration of the pThr sidechain. In contrast, the space released by the absence of pSer cannot be adequately filled by protein sidechains, which creates room for the dihedral of pSer to be more flexible. Although sidechains of the dual loops are mobile, the conserved His, located at the N-terminus of β5, uses the imidazole ring and polar interactions to form stable interactions with residues of loops β4-β5 and β6-β7. For example, Figure 4 (C) shows that His can interact with the conserved Ser85 of loop β4-β5 and Ile104 and Gly108 of loop β6-β7 in Rad53-FHA1. During our MD simulations, the sidechains moved between the two loops, which strengthens the interactions between residues around His (e.g., Ser85 and Thr106) and generates a proper space exclusively for the methyl group. The same movement and enhanced interactions for forming a cavity are observed in multiple 50 ns simulations (data not shown). Therefore, our simulations explain how FHA makes use of the conserved His to stabilize the dual loop and form a structural room to dock the methyl group and discriminate pThr/pSer.

To quantify the flexibility of dihedral rotation and vibration of pSer/pThr, we performed Gibbs entropy calculations. As illustrated in Table 4, the sidechain dihedral entropy increased ~0.7-1.5 kcal/mol after mutation and the configuration entropy of the entire pSer residue increased nearly 2 kcal/mol as compared with pThr. Among all the dihedral angles, the entropy increase is mostly contributed by sidechain dihedrals. Notably, the local entropy increase when pThr is substituted by pSer is only a local effect, and the entropy loss of the whole system with phosphopeptide binding was not computed and compared in this work. The local interaction energy and local entropy calculations offer quantitative comparison for pSer- and pThr-containing peptide binding, and we do not suggest that the binding energy loss may be fully compensated by the local entropy gain in this study.

**Table 4:** Configuration entropy changes

domain	mutated site	mutation	**TΔS**_**phi**_	**TΔS**_**psi**_	**TΔS**_**omega**_	**TΔS**_**sidechain**_	**TΔS**_**total**_
**Rad53-FHA1**	1	**pT**→**pS**	0.18	0.29	0.03	1.39	1.91
**Rad53-FHA1**	1	**pT**→**pS**	0.37	0.37	0.14	1.26	2.16
**Dun1-FHA**	1	**pT**→**pS**	-0.25	-0.08	0.00	0.72	0.45
**Ki67-FHA**	1	**pT**→**pS**	0.25	0.32	0.14	1.50	2.23
**Dun1-FHA**	2	**pT**→**pS**	0.10	-0.01	0.00	1.03	1.12
**Ki67-FHA**	2	**pS**→**pT**	-0.04	-0.06	0.01	-0.47	-0.57
**BRCT**	1	**pS**→**pT**	-0.08	0.01	0.00	0.35	0.28
**WW**	1	**pS**→**pT**	-0.15	-0.01	-0.12	-0.71	-1.01

Configuration entropy changes (kcal/mol) for phi, psi, omega and sidechain dihedrals of phosphoresidue in the wild-type and mutants.

### The second phosphoresidue-binding site of Dun1-FHA and Ki67-FHA

Some FHA domains also show the second phosphoresidue-binding site, and knowing whether the second site can discriminate pThr and pSer is of interest. We therefore studied two diphosphoresidue-recognized FHA domains, Dun1-FHA and Ki67-FHA. Both domains have one pThr binding to the main pThr-binding site, but they also have one more phosphoresidue, pSer or pThr, in the peptide sequences. One main difference between the main pThr and the second phosphoresidue-binding site is that the main pThr-binding site consists of two loops that form a well-defined pocket, whereas the second binding site is located in areas with a single loop. To understand whether the difference contributes to residue specificity, we mutated the phosphoresidue available in the experiment, pThr of Dun1-FHA and pSer of Ki67-FHA to pSer and pThr, respectively.

Overall, the global binding energy calculations show that for the second phosphoresidue-binding site, the mutations worsen binding affinities (see Table 2), but the changes are smaller than the values for the main pThr-binding site. However, the local interaction energy calculations do not show the same trend, and the mutation of Dun1-FHA is preferable. Therefore, the calculations do not directly support that the domain strongly prefers either pThr or pSer in the second phosphoresidue-binding site. The local interaction energy calculations suggest that pSer can have good interactions with the domain, which are contributed mainly from the electrostatic attractions, and by losing the methyl group, the van der Waal interactions are weakened, but not significantly. As illustrated in Figure 2 (B2) and (B3), the second phosphoresidue-binding site in Dun1-FHA uses two Arg residues, Arg62 and Arg64, to recognize pThr or pSer and form multiple H-bonds with the phosphate group. Therefore, the electrostatic attractions are the major driving forces in pThr/pSer binding is not surprising [56]. In addition, both Arg residues are located in one single loop, which is a flexible region of FHA domains, so the protein is freely adjustable to adopt both pThr and pSer. Although the methyl group of pThr forms non-polar attractions with the alkane branch of Arg62 shown in Figure 2 (B2), the binding site does not hold a small pocket when pThr is substituted by pSer (Figure 2 (B3)), because the space is filled by the nearby FHA domain sidechains.

The second phosphoresidue-binding site is located in a single loop, β3-β4, and without spatial constraint, the second site allows the FHA domain to rearrange sidechains to optimize both pThr and pSer binding. We therefore examined changes in local flexibility when a different phosphoresidue stays in this binding site, and we focused on dihedral angles of the phosphate group of pThr and pSer. Both residues do not expressly reveal dynamic motions in either complex, but the most populated angles modeled from our MD simulations shift more than 20° (Figure 3 (C1) (C2)). Although the dihedral angle has only one rotameric states in both cases, pThr has smaller vibration range and the configuration entropy is 0.5-1.0 kcal/mol smaller than pSer, presumably due to a bulkier methyl group. The entropy changes between pThr and pSer is less pronounced in the second phosphoresidue-binding site than in the main one. Moreover, the motions of backbone dihedrals remain the same, which indicates the negligible influence of the mutation.

In conclusion, the main pThr-binding site has a unique feature to recognize pThr, and a special pocket built by linking two loops with the conserved His is reserved for the methyl group of pThr, which plays a crucial role in distinguishing between pThr and pSer. However, the second phosphoresidue binding site is positioned in a single loop near the N-terminus, which uses two Arg residues to recognize a phosphate group but lacks a well structured binding cavity to identify only pThr or pSer. Our simulations show that the protein sidechain of the binding site changes when pThr or pSer binds to the domain. Therefore, a single loop used to provide a phosphoresidue-binding site cannot discriminate pThr/pSer but can bind to both residues. Other domains, such as the WW domain, also use a similar strategy, as discussed in the next section. Of note, although not within the scope of this paper, the promiscuous domain has preferences for selected sequences, and the peptide sequences also play an important role in the phosphopeptide-binding site.

### BRCT repeats and WW domain recognize both pThr and pSer

The BRCT and WW domains are distinct pSer/pThr binding domains. Not all BRCT and WW domains function as phosphopeptide binding modules, but both have a subset that binds to phosphopeptides. Both domains can bind to particular sequences that contain pSer or pThr, but in general, tandem BRCT domains bind stronger to pSer than pThr and WW domains have a preference for pThr preceding a Pro [28,33]. Note that pSer or pThr must be followed by Pro, for pSer/pThr-Pro sequences for binding to WW domains. In contrast to FHA domains, which bind exclusively to pThr-containing peptides, BRCT and WW domains do not recognize solely pThr- or pSer-containing peptides. Although proteins that treat Ser and Thr as similar residues may be common, knowing how both domains have a specific or non-specific pThr/pSer recognition is of interest.

Both global binding energy and local interaction energy calculations suggest that the tandem BRCT domains prefer the pSer- than pThr-containing sequence, although the preference is not strong. The local interaction energies shown in Table 3 suggest that pSer can form a more favorable electrostatic attraction, ~0.5 kcal/mol more negative than that contributed by pThr binding, but the difference is relatively small. In addition, our simulations show that most of the time, the methyl group of pThr does not directly interact with the domain, as demonstrated by a representative complex conformation in Figure 2 (C2). As a result, we see a negligible difference in van der Waal interactions in the local interaction energy calculations (see Table 3), and the electrostatic attractions are the main driving forces to recruit phosphopeptides binding to tandem BRCT domains (Table S2 in Additional file 1). The phospho recognition is through forming interactions with Lys1702 in an α helix and Ser1655 and Gly1656 near the loop, where no small cavity is reserved for the methyl group of pThr. Although the methyl group is not directly involved in binding, the local arrangement of the phosphate group is changed, but the overall flexibility of the phosphate group remains similar (Figure 2 (C)).

Although the WW domains are able to recognize both pSer- and pThr-containing peptides, global binding energy calculations suggest that the domain favors pThr because of the more preferable van der Waals attractions. The trend is in agreement with experimental results [33]. However, the local interaction energy calculations show that the favorable van der Waals attractions are mostly compensated by weaker electrostatic interactions. As shown in Figure 2 (D2) and (D3), two conserved aromatic residues of the domain, Tyr and Trp, create a cavity, but no sidechains of phosphopeptides could nicely fit into the cavity during our simulations. Interestingly, the conserved Pro residue adjacent to the phosphoresidue is clamped by Tyr and Trp, which stays in the cavity and further restricts nearby phosphopeptide conformations. The confined region formed by rings of Tyr, Trp and Pro is conserved regardless of the presence of pSer or pThr (see Figure 2 (D2) and (D3)), which also explains the crucial roles of Pro. Because of the bulky ring conformations, an empty space is observed during the course of the simulations. The empty space can be partially filled by the methyl group of pThr, thus resulting in more favorable van der Waals interactions and a less flexible sidechain while pThr is binding. However, the Pro residue but not the methyl group of pThr primarily occupies the cavity in phosphopeptide recognition. Therefore, the domains do not show significant discrimination between pSer and pThr.

### Comparisons between FHA domains and tandem BRCT repeats and WW domain

FHA domains use the conserved His to bridge two loops, β4-β5 and β6-β7, to construct the main pThr binding, which have a phosphate group binding site and preserve a small pocket nicely fit by the methyl group of pThr (see Figure 4(C)). Without the methyl group, neither pSer binding nor rearranging sidechains of FHA near the methyl binding site can effectively fill the pocket, which results in unsuccessful binding. The second binding site of FHA domains makes use of a single loop to recognize pThr/pSer, and WW and BRCT domains combine a single loop and a nearby α helix (BRCT domains) or β sheet (WW domains) to bind to pThr/pSer. The structures of these phosphoresidue binding sites allow the protein sidechains to be adjustable to both pThr and pSer residues. Notably, although loops are usually considered flexible regions of a protein, the dual loops in the main pThr-binding site of FHA domains show an interaction network between the loops to form a pre-organized binding cavity for pThr (data not shown). Besides the unique features of using dual loops specifically for pThr binding, all other phosphoresidue binding sites share common characteristics that include a binding site comprised of positive-charged residues to form Coulombic attractions with the phosphate group and geometry complementary in the binding surface.

Of note, the binding affinities of phosphopeptide binding to these domains are in general weak, in the micromolar range (see Table 1); therefore, weakened attractions by a few kcal/mol can completely diminish the phosphopeptides binding. Therefore, although substitution of pThr by pSer mainly reduces van der Waals attractions in the main pThr binding site of FHA domains, the pSer-containing peptide cannot form the domain-peptide complex. Different from other phosphopeptide binding sites, sidechain rearrangements cannot bring other attraction forces to compensate for the loss of the van der Waals interactions because of the rigid structure formed by His and the dual loops. In addition to energy calculations, our local entropy calculations suggest that binding pThr to the main pThr-binding site of FHA can reduce the mobility significantly, which indicates stronger attraction and more geometry confinement. However, the entropy changes between pThr and pSer binding to other domains show smaller differences, which suggests that the system retains a similar dynamic behavior that may help balance energy loss by gaining other attractions.

### Biological implication

Modular domains are common regulators in important biological processes. This work studied three important domains for DNA damage responses, FHA, BRCT and WW domains, all with a phosphopeptide binding site to relay the damage signal and trigger further repair. The specific peptide can be recognized by three different proteins: a protein kinase to phosphorylate Ser/Thr, a modular domain that binds to the phosphopeptide for a downstream process, and a phosphatase to dephosphorylate the phosphoresidue. Kinases involved in the DNA damage response, ATM and ATR, can phosphorylate both Ser and Thr of a substrate. Similar to kinases, phosphatases work for both pThr and pSer [57], and most phosphodomains can also bind to both pThr- and pSer-containing peptides. FHA domains have evolved a simple but remarkable mechanism to specifically recognize pThr to further select particular partners after kinase phosphorylation. For example, a binding partner of FHA domains, the SCD protein family, contains rich Thr-glutamine (TQ) and Ser-glutamine (SQ) repeat motifs [58]. Although the kinase phosphorylates both Thr and Ser, Rad53-FHA1 can bind only to regions that have pThr. How binding to the particular pThr region triggers further responses is unclear, but the pThr-only recognition may play a role in regulation. The mechanism contributing to pThr binding brings insights into how modular domains differentiate pThr and pSer or recognize both residues. The information aids in the design and discovery of phosphopeptides to access the cellular function of the domain-containing proteins. The rigid dual loops centered on the conserved His in the main pThr binding site of FHA domains may be applied to protein engineering that may need to recognize small functional groups.

## Conclusions

In this study, we performed dynamic-guided process for FHA, BRCT and WW domain-peptide structures. The components of detailed interaction energies were calculated by MM-PBSA/MM-GBSA method. The main pThr-binding cavity is identical in four different FHA complexes. Our results reveal FHA domain uses the conserved His residue to define a dual loops structure which shows strong favor for pThr because of the geometry of methyl group embedded in deep binding pocket nicely. The dynamics simulations, energy and entropy calculations indicate that the phosphoresidue binding site of FHA is highly suited to pThr, and small changes of pThr to pSer can diminish the domain-peptide interactions due to the pre-organized binding cavity. On the other hand, BRCT repeats and WW domain utilize the combination a single loop with α-helix or β-sheet which allows effectively sidechain rearrangement to accept both pSer and pThr. The results highlight broader implications in recognition pathway of kinase/phosphotase and also help to engineer proteins and design peptide inhibitors.

## Authors' contributions

YMH and CAC carried out MD, participated in post-analysis and drafted manuscript. All authors read and approved the final manuscript.

## Supplementary Material

Additional file 1 **Table S1**: MM-PBSA energy calculations for each seed **Table S2**: Local interaction energy calculations (MM-GBSA) for wild-type and mutated MD trajectory **Table S3**: List of residues selected around phosphoresidue **Figure S1**: RMSD plot of Rad53-FHA1 **Figure S2**: Detailed illustration of pThr/pSer peptide binding to FHA Click here for file
