# Supplementary material for: Mechanism of PhosphoThreonine/Serine Recognition and Specificity for Modular Domains from All-atom Molecular Dynamics
Source: BMC Biophys. 2011 May 25;4:12. doi: 10.1186/2046-1682-4-12 (PMC3146460; doi:10.1186/2046-1682-4-12)

Mechanism of PhosphoThreonine/Serine Recognition and Specificity for Modular Domains from All-Atom Molecular Dynamics

Yu-ming M. Huang[1] *and Chia-en A. Chang*[1,*]

[1] Department of Chemistry, University of California, Riverside, Riverside, California, 92521, USA

* Corresponding author: Dr. Chia-en Chang

Email: chiaenc@ucr.edu

Telephone: (951) 827-7263

Fax: (951) 827-2040

Table S1: MM-PBSA energy calculations for each seed. The average energies are in red. The notations are the same as those in Table 2.

1: Rad53-FHA1 (1g6g)

2: Rad53-FHA1 (1k3q)

3: Dun1-FHA (2jql)

4: Ki67-FHA (2aff)

5: BRCT (1t2v)

6: WW (1f8a)

7: Rad53-FHA1 (1g6g): first pT→pS

8: Rad53-FHA1 (1k3q): first pT→pS

9: Dun1-FHA (2jql): first pT→pS

10: Ki67-FHA (2aff): first pT→pS

11: BRCT (1t2v): pS→pT

12: WW (1f8a): pS→pT

13: Dun1-FHA(2jql): second pT→pS

14: Ki67-FHA(2aff): second pS→pT

No	seed	ΔU_{VDW}	ΔU_{Coul}	ΔW_{PB}	ΔE_{ele}	$\Delta E_{tot\text{-}np}$	ΔW_{np}	ΔE_{tot}
**1**	1	-35.36	-416.07	413.48	-2.59	-37.94	25.87	-12.07
	2	-35.46	-451.82	444.94	-6.88	-42.34	26.75	-15.59
	3	-34.21	-473.44	471.93	-1.51	-35.72	27.45	-8.27
	4	-32.28	-463.21	457.37	-5.84	-38.12	24.80	-13.32
	5	-31.70	-418.69	415.19	-3.49	-35.19	24.21	-10.98
	**Avg**	**-33.80±5.1**	**-444.65±36.0**	**440.58±34.7**	**-4.06±10.5**	**-37.86±8.8**	**25.82±2.1**	**-12.05±9.5**
**2**	1	-32.20	-385.17	403.95	18.78	-13.42	25.96	12.54
	2	-34.29	-424.26	446.37	22.11	-12.18	27.97	15.79
	3	-33.81	-366.87	385.02	18.15	-15.66	26.55	10.89
	4	-32.78	-381.60	409.25	27.64	-5.14	27.41	22.27
	5	-32.99	-421.85	443.39	21.54	-11.45	27.40	15.95
	**Avg**	**-33.21±4.3**	**-395.95±42.5**	**417.60±40.8**	**21.64±9.6**	**-11.57±8.8**	**27.06±1.7**	**15.49±9.3**
**3**	1	-32.07	-583.67	597.75	14.09	-17.98	22.56	4.58
	2	-29.29	-649.92	660.40	10.49	-18.80	21.82	3.01
	3	-26.83	-717.66	722.65	4.99	-21.84	23.57	1.73
	4	-32.65	-619.92	641.77	21.84	-10.81	23.52	12.71
	5	-29.84	-615.44	631.18	15.74	-14.10	23.24	9.14
	**Avg**	**-30.14±6.3**	**-637.32±60.6**	**650.75±55.4**	**13.43±13.6**	**-16.71±11.6**	**22.94±2.1**	**6.23±12.3**
**4**	1	-128.26	-1175.64	1190.01	14.37	-113.89	76.25	-37.63
	2	-122.86	-1187.87	1195.24	7.38	-115.48	77.00	-38.49
	3	-130.70	-1189.37	1206.24	16.87	-113.83	76.40	-37.44
	4	-126.47	-1201.62	1204.86	3.24	-123.23	76.95	-46.28
	5	-124.78	-1205.51	1212.30	6.79	-117.99	75.78	-42.20
	**Avg**	**-126.61±7.6**	**-1192.00±51.6**	**1201.73±49.5**	**9.73±17.7**	**-116.88±16.6**	**76.48±2.4**	**-40.41±17.0**
**5**	1	-39.22	-96.74	121.80	25.07	-14.15	30.25	16.10
	2	-38.56	-57.59	81.52	23.92	-14.64	31.04	16.40
	3	-37.69	-104.55	140.11	35.56	-2.14	30.93	28.79
	4	-39.38	-72.71	106.52	33.81	-5.57	31.24	25.68
	5	-38.74	-88.97	124.74	35.77	-2.97	31.01	28.04
	**Avg**	**-38.72±4.4**	**-84.11±32.3**	**114.94±31.9**	**30.83±12.2**	**-7.89±11.2**	**30.90±1.5**	**23.00±11.8**
**6**	1	-31.21	-615.18	602.06	-13.12	-44.33	19.47	-24.85
	2	-31.02	-579.70	564.03	-15.67	-46.69	19.95	-26.74
	3	-29.18	-621.11	605.87	-15.24	-44.41	18.87	-25.54
	4	-31.24	-611.17	595.68	-15.49	-46.73	19.09	-27.64
	5	-30.80	-596.14	582.72	-13.42	-44.22	18.79	-25.43
	**Avg**	**-30.69±4.8**	**-604.66±29.4**	**590.07±28.1**	**-14.59±8.5**	**-45.28±7.1**	**19.24±1.1**	**-26.04±7.4**
**7**	1	-33.89	-502.37	507.49	5.13	-28.76	28.70	-0.06
	2	-27.00	-503.92	494.06	-9.86	-36.86	23.57	-13.30
	3	-30.74	-435.35	441.42	6.06	-24.68	26.07	1.39
	4	-34.80	-439.41	445.01	5.60	-29.20	28.83	-0.36
	5	-30.06	-513.89	504.10	-9.80	-39.86	25.66	-14.19
	**Avg**	**-31.30±6.3**	**-478.99±47.0**	**478.42±41.4**	**-0.57±13.0**	**-31.87±10.7**	**26.57±3.0**	**-5.31±11.8**

**8**	1	-32.13	-347.14	379.05	31.91	-0.23	26.91	26.69
	2	-33.99	-441.80	459.08	17.28	-16.71	28.25	11.54
	3	-32.62	-415.84	440.43	24.59	-8.04	27.92	19.88
	4	-33.36	-414.03	436.73	22.70	-10.67	28.51	17.84
	5	-31.76	-429.66	453.08	23.42	-8.34	27.29	18.95
	**Avg**	**-32.77±5.0**	**-409.69±55.7**	**433.67±50.3**	**23.98±10.6**	**-8.79±10.1**	**27.77±1.7**	**18.98±10.1**
**9**	1	-30.28	-677.72	698.46	20.74	-9.54	24.13	14.60
	2	-28.17	-663.89	681.59	17.70	-10.46	24.69	14.23
	3	-38.33	-655.79	680.31	24.52	-13.81	27.53	13.72
	4	-28.39	-634.57	648.39	13.82	-14.56	23.44	8.87
	5	-38.66	-623.13	639.23	16.09	-22.57	26.63	4.06
	**Avg**	**-32.76±8.0**	**-651.02±36.0**	**669.59±35.9**	**18.58±12.6**	**-14.19±11.5**	**25.28±2.3**	**11.10±11.6**
**10**	1	-119.40	-1181.88	1193.27	11.39	-108.00	74.38	-33.62
	2	-120.73	-1235.41	1246.02	10.61	-110.11	75.84	-34.27
	3	-121.16	-1156.96	1166.28	9.32	-111.84	74.29	-37.55
	4	-123.83	-1212.77	1221.64	8.87	-114.96	76.95	-38.01
	5	-126.46	-1216.84	1220.37	3.53	-122.93	76.74	-46.19
	**Avg**	**-122.32±7.1**	**-1200.77±61.4**	**1209.52±58.8**	**8.74±16.0**	**-113.57±16.2**	**75.64±2.6**	**-37.93±16.5**
**11**	1	-38.47	-32.77	62.87	30.10	-8.37	32.65	24.29
	2	-39.44	-66.33	103.30	36.97	-2.48	30.31	27.83
	3	-42.64	-29.51	56.95	27.44	-15.20	30.90	15.70
	4	-43.22	-98.60	140.40	41.80	-1.42	32.33	30.91
	5	-46.49	-49.49	92.82	43.34	-3.15	32.48	29.33
	**Avg**	**-42.05±5.3**	**-55.34±38.1**	**91.27±42.7**	**35.93±11.8**	**-6.12±10.9**	**31.74±1.5**	**25.61±11.3**
**12**	1	-32.57	-619.58	606.44	-13.14	-45.72	19.54	-26.17
	2	-32.38	-612.40	592.96	-19.44	-51.83	19.20	-32.63
	3	-32.01	-705.68	687.98	-17.70	-49.71	19.70	-30.02
	4	-31.75	-658.08	640.31	-17.77	-49.52	18.80	-30.72
	5	-29.88	-626.29	606.59	-19.70	-49.58	18.92	-30.66
	**Avg**	**-31.72±5.2**	**-644.41±59.6**	**626.86±58.4**	**-17.55±11.2**	**-49.27±9.6**	**19.23±1.1**	**-30.04±9.9**
**13**	1	-38.99	-717.35	736.55	19.20	-19.78	27.51	7.73
	2	-35.05	-663.17	689.86	26.69	-8.36	25.38	17.02
	3	-25.91	-683.02	693.81	10.79	-15.12	21.78	6.66
	4	-30.32	-712.37	723.49	11.12	-19.20	24.58	5.38
	5	-33.11	-707.20	727.00	19.79	-13.31	25.22	11.90
	**Avg**	**-32.67±7.3**	**-696.62±48.9**	**714.14±42.5**	**17.52±15.5**	**-15.16±13.7**	**24.89±3.1**	**9.74±14.4**
**14**	1	-125.42	-1233.21	1242.42	9.21	-116.21	76.46	-39.75
	2	-130.62	-1120.26	1131.75	11.49	-119.13	79.62	-39.51
	3	-119.02	-1147.45	1159.00	11.54	-107.48	74.94	-32.54
	4	-129.12	-1255.76	1265.38	9.62	-119.50	80.32	-39.18
	5	-124.19	-1104.48	1109.34	4.86	-119.33	72.83	-46.50
	**Avg**	**-125.67±8.3**	**-1172.23±86.2**	**1181.58±85.3**	**9.34±15.5**	**-116.33±14.4**	**76.83±3.9**	**-39.50±14.9**

Table S2: Local interaction energy calculations (MM-GBSA) for wild-type and mutated MD trajectory. The notations are the same as those in Table 2.

domain	ΔU_{VDW}	ΔU_{Coul}	ΔW_{PB}	ΔE_{ele}	ΔE_{tot-np}	ΔW_{np}	ΔE_{tot}
Rad53-FHA1 first pT	-19.41±4.0	-728.15±21.5	610.39±14.0	-117.76±10.5	-137.17±8.8	-4.09±0.2	-141.26±8.9
Rad53-FHA1 first pT	-17.36±3.6	-458.72±27.2	392.53±19.8	-66.19±10.5	-83.55±9.2	-3.00±0.2	-86.55±3.6
Dun1-FHA first pT	-15.77±4.8	-265.88±21.7	197.81±14.8	-68.08±9.6	-83.85±8.1	-2.60±0.3	-86.45±8.3
Dun1-FHA second pT	-9.26±3.7	-215.82±18.6	180.43±12.3	-35.39±9.3	-44.64±9.7	-1.50±0.3	-46.14±9.9
Ki67-FHA first pT	-15.27±3.0	-230.06±10.6	174.89±7.1	-55.17±6.2	-70.44±5.1	-2.67±0.1	-73.11±5.1
Ki67-FHA second pS	-85.12±1.1	-137.09±10.3	129.11±8.2	-7.93±2.9	-13.05±3.2	-0.74±0.1	-13.79±3.3
BRCT pS	-7.93±3.0	-313.36±16.9	251.33±13.9	-62.04±7.1	-69.96±5.9	-2.02±0.2	-71.99±6.0
WW pS	-21.90±4.1	-537.42±17.8	442.31±13.9	-95.11±9.1	-117.01±7.1	-3.26±0.1	-120.27±7.1
Rad53-FHA1 first pS	-16.29±4.6	-736.52±30.7	616.10±19.6	-120.42±13.4	-136.71±11.0	-4.05±0.2	-140.76±9.5
Rad53-FHA1 first pS	-17.12±4.2	-468.37±29.5	403.47±20.1	-64.90±12.8	-82.02±12.0	-3.12±0.3	-85.14±12.2
Dun1-FHA first pS	-13.47±5.8	-262.87±16.5	197.09±9.9	-65.79±10.1	-79.25±7.9	-2.53±0.3	-81.78±8.0
Ki67-FHA first pS	-12.05±3.2	-227.80±11.1	173.21±7.7	-54.59±6.1	-66.64±4.8	-2.52±0.1	-69.16±4.8
BRCT pT	-7.86±3.0	-306.19±21.0	244.57±16.8	-61.62±7.0	-69.49±5.9	-1.93±0.1	-71.42±6.0
WW pT	-23.75±4.5	-535.75±19.8	442.42±16.7	-93.33±10.2	-117.08±8.5	-3.21±0.2	-120.29±8.6
Dun1-FHA second pS	-8.76±4.6	-217.13±33.7	180.17±22.6	-36.96±13.0	-45.72±14.7	-1.50±0.4	-47.22±15.1
Ki67-FHA second pT	-4.41±1.6	-13.76±11.3	127.13±8.3	-6.62±3.8	-11.04±4.2	-0.64±0.2	-11.68±4.3

Table S3: List of residues selected around phosphoresidue. Blue and red indicate the residues from protein and peptide, respectively.

domain	PDb ID	select residues
Rad53-FHA1 first pT→pS	1G6G	R70, S82, R83, L84, S85, N86, K87, H88, S105, T106, N107<br>E3, V4, pT5, E6, A7, D8
Rad53-FHA1 first pT→pS	1K3Q	R70, I81, S82, R83, L84, S85, N86, S105, T106, N107<br>E167, V168, pT169, E170, A171
Dun1-FHA first pT→pS	2JQL	R62, D74, I75, S76, T77, F78, R104<br>Q6, P7, pT8, Q9, Q10
Dun1-FHA second pT→pS	2JQL	R62, S63, R64<br>N3, I4, pT5, Q6, P7
Ki67-FHA first pT→pS	2AFF	R31, V43, V44, S45, S65, T66, N67<br>G232, P233, pT234, P235, V236
Ki67-FHA second pS→pT	2AFF	R31, G32<br>D229, pS230, Q231
BRCT pS→pT	1T2V	V1654, S1655, G1656, L1657, N1678, L1679, T1700, L1701, K1702<br>P5, I6, pS7, Q8, V9
WW pS→pT	1F8A	R18, M19, S20, R21, S22, S23, G24, R25, V26, T27, S36, E37, W38<br>P172, T173, pS174, P175, S176

Figure S1: RMSD plot of Rad53-FHA1. Each color presents each seed. Wild-type and mutated simulation are shown as (a) and (b), respectively.

(a)

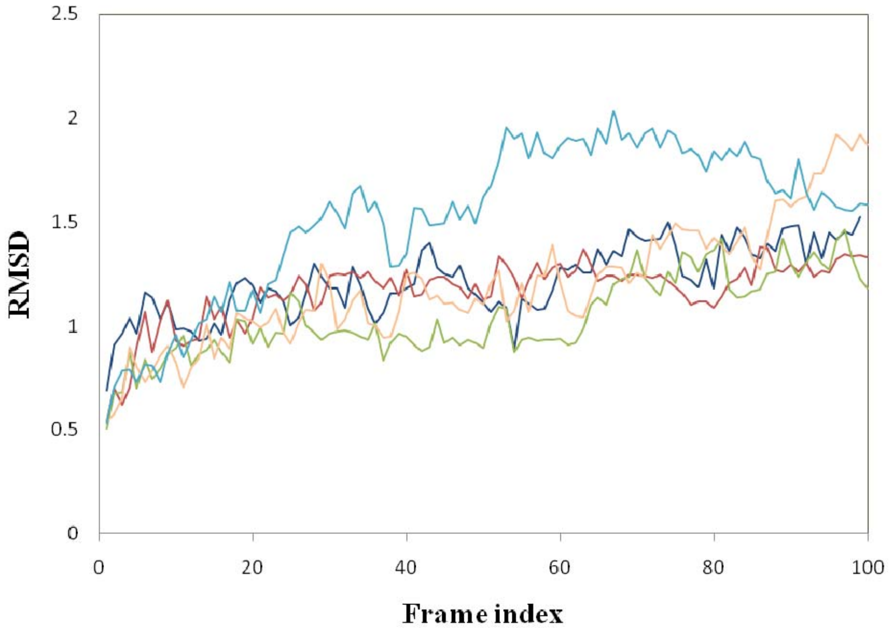

(b)

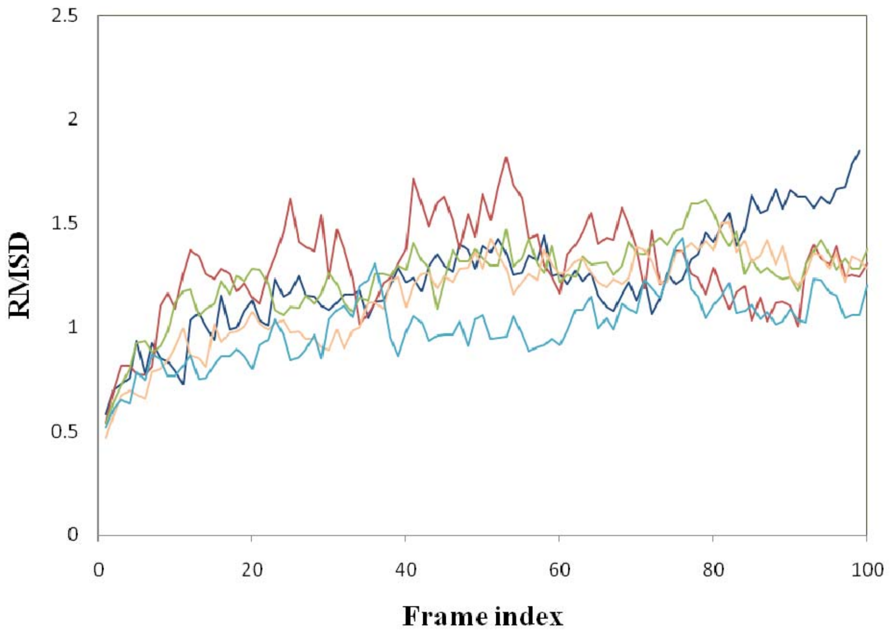

Figure S2: Detailed illustration of pThr/pSer peptide binding to FHA. (A) the main binding site of Rad53-FHA1, (B) the second binding site of Dun1-FHA, (C) the main binding site of Ki67-FHA and (D) the second binding site of Ki67-FHA. The binding areas are circled in the left column (1). The notations are the same in Figure 2.

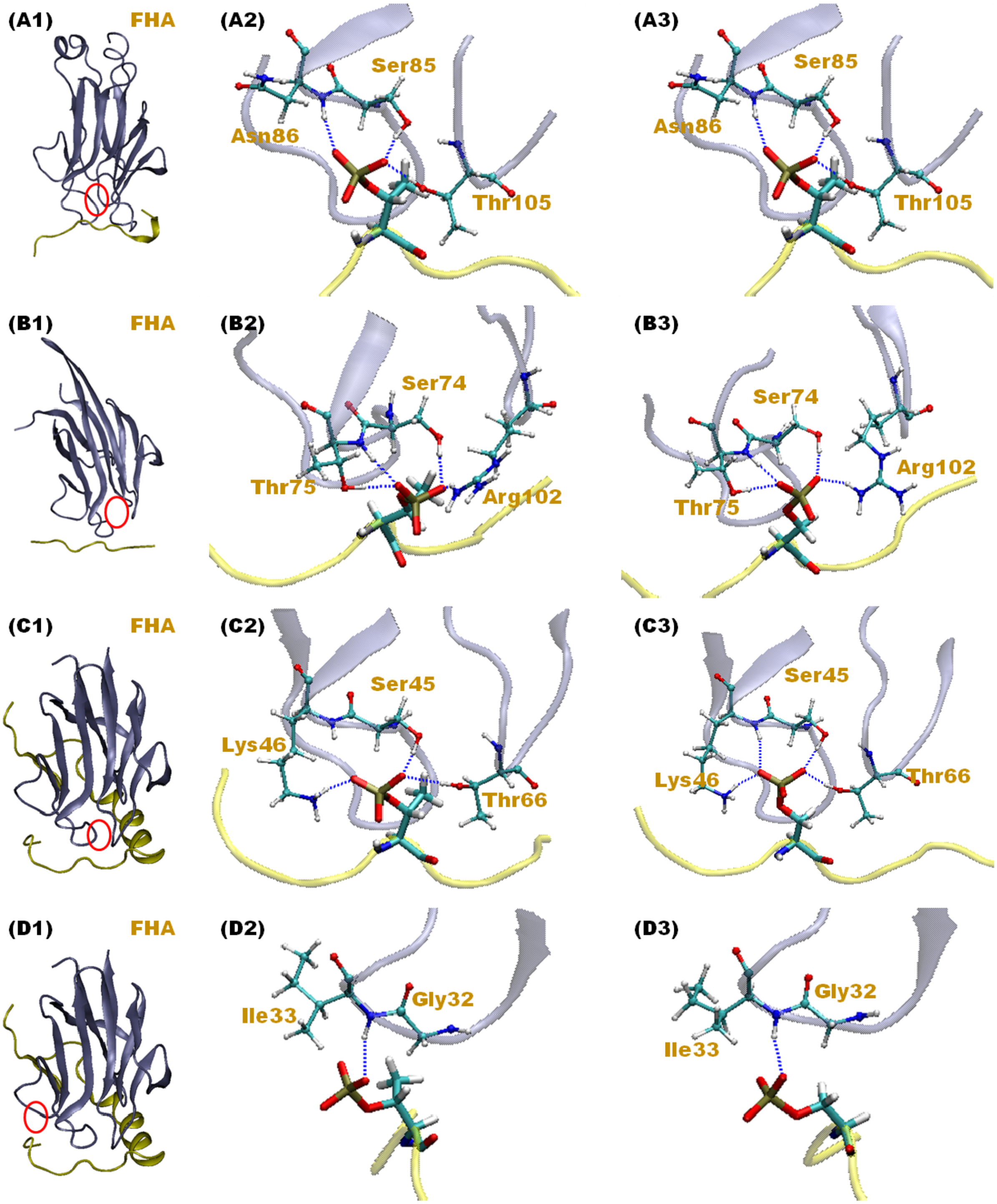

Supplement: Additional file 1 — Table S1: MM-PBSA energy calculations for each seed Table S2: Local interaction energy calculations (MM-GBSA) for wild-type and mutated MD trajectory Table S3: List of residues selected around phosphoresidue Figure S1: RMSD plot of Rad53-FHA1 Figure S2: Detailed illustration of pThr/pSer peptide binding to FHA [file 2046-1682-4-12-S1.PDF]
